# Physical Health and Psychological Outcomes in Adult Patients with Long-Bone Fracture Non-Unions: Evidence Today

**DOI:** 10.3390/jcm8111998

**Published:** 2019-11-15

**Authors:** Louise Johnson, Emily Igoe, George Kleftouris, Ioannis V. Papachristos, Costas Papakostidis, Peter V. Giannoudis

**Affiliations:** 1 Leeds Teaching Hospitals NHS Trust, Leeds Major Trauma Centre, Leeds General Infirmary, Leeds LS1 3EX, UK; emilyjaneigoe@hotmail.com (E.I.); george.kleftouris@nhs.net (G.K.); 2 Department of Trauma and Orthopaedic Surgery, Leeds Teaching Hospitals NHS Trust, Leeds Major Trauma Centre, Leeds General Infirmary, Leeds LS1 3EX, UK; ioannispapachristos@nhs.net; 3 Orthopaedic Department, Limassol General Hospital, PO BOX 56060 Limassol, Cyprus; costaspapakostidis@gmail.com; 4 Academic Department of Trauma & Orthopaedics, School of Medicine, University of Leeds, NIHR Leeds Biomedical Research Centre, Chapel Allerton Hospital, Leeds LS2 9JT, UK; peter.giannoudis@nhs.net

**Keywords:** fracture non-union, systematic review, physical health, psychological outcomes

## Abstract

Background: Research has suggested that bone fractures can hinder the health status of patients’ life. However, limited research has examined the impact that the healing process of a fracture has on the physical health and psychological state of individuals, particularly in considering the short- and long-term impact of having a fracture that fails to heal and drops into a non-union. The aim of this systematic review is to better understand the impact of fracture non-union to physical health and to respective psychological outcomes. Methods: Electronic databases ‘PubMed’, ‘Cochrane’, ‘PsycInfo’, ‘Medline’, ‘Embase’, ‘Web of Science’, and ‘CINAHL’ were used. Search terms used were nonunion OR non-union OR “non union” OR “long bone” OR “delayed union” AND “quality of life” OR qol OR depression OR anxiety OR psycholog* OR PTSD OR “post-traumatic stress disorder”. Studies published in the years 1995 to 2018 were included. Two independent reviewers carried out screening and data extraction. Studies were included if (1) participants were adult (human) patients with a traumatic non-union secondary to fracture/s; (2) outcomes measured included physical health and psychological wellbeing (e.g., PTSD, psychological trauma, depression, anxiety, etc.). Studies received emphasis if they compared those outcomes between: (1) The “non-union” group to a normative, matched population and (2) the “non-union group” to the same group after union was achieved. However, studies that did not use comparison groups were also included. Results: Out of the 1896 papers identified from our thorough literature search, 13 met the inclusion criteria. Quality assessment was done by the Methodological Index for Non-Randomized Studies (MINORS). Findings suggested that non-unions had a detrimental impact on physical health, and psychological difficulties often after recovery. Conclusions: Patients who experience a long bone non-union are at risk of greater psychological distress and lower physical health status. There is a need for early identification of psychological distress in patients with fracture non-unions and psychological provision should become part of the available treatment.

## 1. Introduction

According to the current definition from the European Society of Tissue Regeneration in Orthopedics and Traumatology (ESTROT), a non-union is defined as a fracture that does not heal without a further intervention—independent of the length of the previous treatment [1]. NICE guidelines furthermore set a time frame of 9 months of failure to achieve healing for a fracture to be considered as a non-union [2]. FDA rules are in agreement with the above timeframe [3]. 

It is estimated that around 5%–10% of fractures will develop a non-union [4]. The current method of treatment varies in individuals, but when surgery is the chosen method, internal or external fixation and bone grafting are commonly used (according to NICE [5]). Factors that increase risk for developing a non-union can be classified as patient-dependent, such as older age, medical comorbidities (diabetes mellitus, vascular disease), smoking, NSAIDs use, nutritional deficiency and genetic or metabolic disorders, as well as patient–independent factors like degree of comminution, infection, fracture site (for example a base of 5th metatarsal fracture), bone loss, open fracture and quality of surgical treatment [3]. This complication does not come without a cost. Kanakaris and Giannoudis estimated that in the best-case scenario, the overall cost per patient suffering from a fracture non-union is around £15,566 for a humeral pseudarthrosis with the cost increasing to £16,330 for a tibial non-union and all the way up to £17,000 for a femoral pseudarthrosis [6]. The economic implications of this condition, suggests the importance of research in order to gain insight into this burden, which includes the ‘intangible costs’ of the impact on patient physical health state and ‘psychosocial parameters’.

Despite the limited investigation into the physical health/psychosocial functioning of patients suffering from fracture non-unions, the available research has suggested that pseudarthroses are detrimental to patients’ overall physical health and quality of life. Some studies have demonstrated this impact at the point of treatment [7,8], and others have investigated and demonstrated this following patients’ recovery from their non-union [9,10]. Despite these findings, a systematic review of the impact of non-unions on adults’ physical health state, and the relevant psychological impact, has not yet been carried out. Therefore, we conducted a systematic review of current literature trying to ascertain the impact of non-unions on adult patients’ physical health state and on their psychological wellbeing.

## 2. Materials and Methods

### 2.1. Literature Search and Data Extraction

The systematic review is registered in Prospero (registration number CRD42016051474) and followed the PRISMA (Preferred Reporting Items for Systematic reviews and Meta-Analyses) guidelines [11]. At the start of our project, a written protocol was established consisting of clearly defined eligibility criteria, as well as criteria for further subgroup and sensitivity analyses. 

#### 2.1.1. Inclusion Criteria

We used the following inclusion criteria in the PICO format to identify eligible studies:

- *Participants*—adult (human) patients over the age of 18 years with diaphyseal long bone fracture non-union(s). Other anatomical sites and non-diaphyseal fracture types were excluded. Eligible studies were those written in English and published after 1995.
- *Intervention*—no particular intervention needed.
- *Comparison*—to the situation after union was achieved or to matched population norms.
- *Outcome*—physical health measures and psychological wellbeing measures (e.g., Post-Traumatic Stress Disorder (PTSD), psychological trauma, depression, anxiety, etc.).

#### 2.1.2. Exclusion Criteria

Studies dealing with non-unions in other anatomical sites than the diaphysis of the long bones, those including patients younger than 18 years-old, experimental studies, studies carried out before 1995 or not written in English were excluded.

Electronic databases ‘PubMed’, ‘Cochrane’, ‘PsycInfo’, ‘Medline’, ‘Embase’, ‘Web of Science’, and ‘CINAHL’ were used. The search was structured to combine the following terms: nonunion OR non-union OR “non union” OR “long bone” OR “delayed union” AND “quality of life” OR qol OR depression OR anxiety OR psycholog* OR PTSD OR “post-traumatic stress disorder”. Studies published in the years 1995 to 2018 were included in the search. The last search was conducted in June 2018. The records identified during the search were screened by two reviewers (EI and GK), at both title and abstract. After excluding all irrelevant articles, based on their title or abstract, the full text of the remaining potentially eligible studies was obtained and evaluated against the eligibility criteria. Any disagreement between the reviewers was resolved by discussion. Extracted data were tabulated on a predefined excel spreadsheet and included: type of study (RCT, cohort study, case series), sample size, location of non-union, fixation device used, and timing of questionnaire administration from point of injury/end of technique/from non-union diagnosis. Neither the authors’ names nor the details of institutions were masked during the search process in order to avoid duplication of data. In addition, the references of all included papers and relevant review articles were screened for potentially eligible studies. No grey literature search was carried out. If there was any information needed in a study that could not be found in the manuscript, the corresponding author was contacted.

### 2.2. Methodological Quality Assessment

The Methodological Index for Non-Randomised Studies (MINORS) was used to assess the methodological quality of included studies [12]. This is a validated instrument to assess the methodological quality of observational primary studies. It consists of 12 items, of which, the first subscale of 8 items are related to non-comparative studies, whereas the last 4 items constitute additional criteria for comparative studies. As the maximum item score is 2, the ideal global score would be 16 for the non-comparative studies and 24 for the comparative studies. Two independently working assessors carried out the quality assessment (LJ, GK). Any disagreement between them was resolved by consensus.

### 2.3. Statistical Analysis

The mean difference along with respective 95% CIs was used to summarize continuous outcomes of interest. Statistical heterogeneity was tested with both Cochran’s Q test [13] and Higgins I^2^ test [14]. For the former, statistical significance was set at 0.1 (as the Q test is characterized by low sensitivity for detecting heterogeneity). As for the latter, an I^2^ value greater than 50% was thought to represent significant heterogeneity.

The RevMan (5.3) software (Review Manager, The Nordic Cochrane Centre, Copenhagen, Denmark) was utilized to calculate the pooled estimate of effects size for the various outcomes of interest, and the degree of statistical heterogeneity present. For the pooling process, the Inverse Variance (IV) statistical method was used and an either fixed or random effects model, depending on the absence or presence of significant statistical heterogeneity, accordingly. The results of pooling were expressed graphically as forest plots. Furthermore, the potential presence of publication bias was investigated by generating funnel plots.

#### 2.3.1. Subgroup Analysis

During the creation of the study protocol we predetermined the following subgroups, based on the anatomical location of the non-union: (i) tibial non-unions; (ii) femoral non-unions. The purpose of subgroup analysis was to explore the impact of the specific anatomical site of the non-union on the physical health state. 

#### 2.3.2. Sensitivity Analysis

The criteria of sensitivity analysis were set a priori at the inception of the study protocol and included studies of poor quality, dubious eligibility or grossly outlying results. The idea was to repeat the pooling process after excluding studies fulfilling the above criteria. Should the above process not produce materially different results compared to the original ones, our confidence on the robustness of the results of our study would increase. 

## 3. Results

### 3.1. Study Selection Procedure

**Figure 1:**
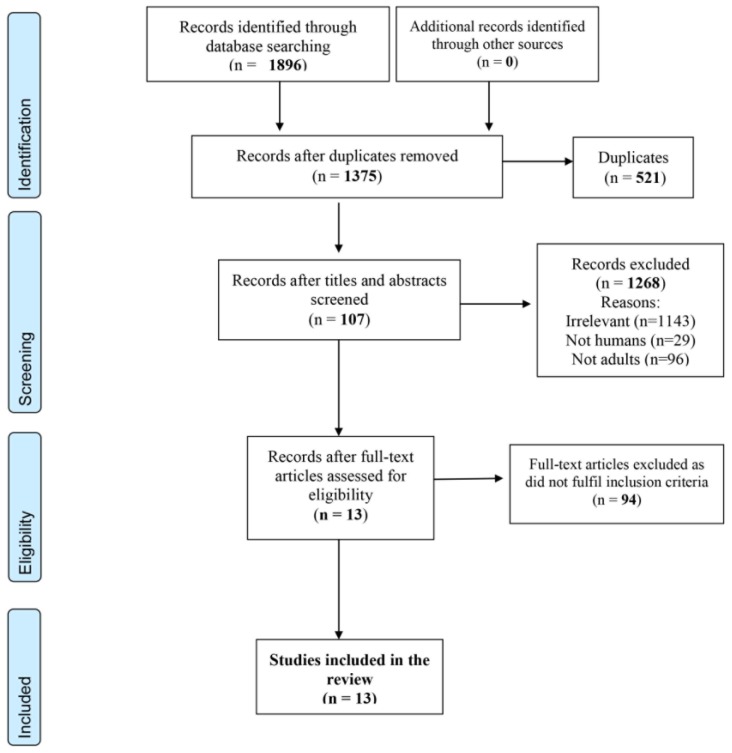
PRISMA 2009 Flow Diagram.

Our thorough literature search identified 1896 reports. After removing 521 duplicates, 1375 remained and were screened on the basis of their title and abstracts. Following our exclusion criteria, we excluded 1268 and the resulting 107 papers were further screened against our inclusion criteria. Eventually, 13 papers were included in our review [7,8,9,10,15,16,17,18,19,20,21,22,23]. All 13 studies were considered for qualitative analysis and seven of them for quantitative analysis [8,9,17,19,20,22,23] (Figure 1).

### 3.2. Characteristics of Included Studies

Nearly half of the included studies (six) came from United States of America [8,11,13,16,17,18], four from Germany [8,10,19,23] and the rest each one from Canada [15], Greece [20], and Australia [7]. Five of them have been published by the same team: three by Brinker et al. (USA) in different years (2007, 2013 and 2017) [8,17,22] and two by Moghaddam et al. (Germany), in 2015 and 2017 [19,23]. Despite originating from the same team, the papers are referring to different patient samples, different anatomical sites and different study timeframes. The list of included studies along with their demographic and baseline characteristics are illustrated in Table 1. In most studies (nine) [7,8,9,10,15,19,20,21,22], males outnumbered females and in half of them (four) [7,10,15,19] the number of females was less than one-third of the number of males. Two studies included more females than males [17,18], one study had an equal sex distribution [23] and in one, this is unknown [16].

Six studies (46%) included only tibial non-unions [8,10,15,17,18,19], two (15%) included only femoral non-unions [22,23], five (38%) studies included both tibial and femoral non-unions [7,9,16,20,21] and two included upper- and lower-limb non-unions [20,21].

Nine studies (69%) included aseptic and septic non-unions [8,10,16,17,19,20,21,22,23], 2 (15%) studies included only aseptic non-unions [9,18], one study included only septic non-unions [15] and one study did not define [7].

Only four out of 13 (30%) mentioned the mechanism of injury [7,10,15,23]. However, no correlation to the physical health state outcomes was made. Type of fracture (open/closed) was identified in eight [7,8,10,15,17,19,22,23] and also biologic type of non-union (hypertrophic/oligotrophic/atrophic) was evident in only three [10,20,23].

No randomised control trials were identified. There were seven prospective cohort studies [8,16,17,18,19,20,23] and six retrospective cohort studies [7,9,10,15,21,22]. Details of the design of the component studies and their outcome measures are summarized in Table 2. Data on both Table 1 and Table 2 reflect the potential presence of clinical heterogeneity across the primary studies.

### 3.3. Methodological Quality of Included Studies

The MINORS score ranged from 9 to 20 across primary studies. Three studies lacked a comparator [17,18,20]. Their quality score ranged from 9 to 12, (ideal global score 16). For the remaining 10 comparative studies [7,8,9,10,11,12,13,14,15,16,19,21,22,23], the quality score ranged between 10 and 20 (ideal global score: 24). The main reasons for low scoring among included studies were lack of power analysis and calculation of appropriate sample size, non-blinded assessment of studies’ end-points, and the use of non-contemporary comparison groups. 

### 3.4. Publication Bias

**Figure 2:**
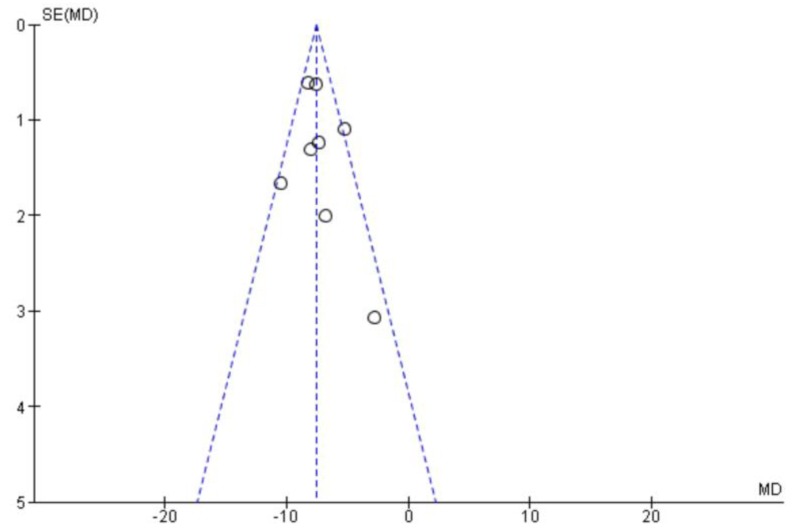
Funnel plot (publication bias).

The potential presence of publication bias was investigated by generating funnel plots for the main outcomes of interest. The distribution of data points in these graphs was symmetrical, indicating that the presence of publication bias was unlikely (Figure 2). Furthermore, all our results were statistically significant and within very narrow 95% CIs. Thus, it is highly unlikely to have missed reports that would have substantially altered our findings. 

### 3.5. Physical Health State Assessment

The majority of studies (nine; 69%) [7,8,9,17,18,19,20,22,23] assessed physical health state using the 12-item short form survey (SF-12) (physical and mental components), four studies [8,18,21,22] used the ‘Time Trade Off’ tool, and three studies [10,15,16] used the 36-item short form survey (SF-36). Other tools used included the 5-Level EQ-5D) [9], and the Western Ontario and McMaster Universities Osteoarthritis Index (WOMAC) [15]. Only one study [9] investigated the psychological impact using the Hospital anxiety and depression score (HADS) and the impact of event scale (IES). Two studies assessed physical health state in patients who failed to progress to bony union following a long-bone fracture [8,21], five assessed QoL only after the intervention [7,9,10,15,22] and six measured it both before and after intervention [16,17,18,19,20,23]. There was variation in the timing of measuring physical health state outcomes; in group of patients whose QoL was assessed postoperatively, this was done either up to 1 year after the revision surgery [7,19,20,23] or during the last follow-up visit (average follow up ranged from 3 to 5 years) [1,10,15,16,18]. Six studies compared the physical health state outcomes between patients and normal population [8,10,15,16,21,22], whereas in three of them [8,21,22] comparison to patients with other orthopaedic or chronic medical conditions was added. Two studies compared outcomes with patients having uneventful fracture healing [7,9]. In five studies, the outcome was compared between pre- and post- intervention for the same group of patients [17,18,19,20,23], whereas in two studies, the scores between different treatment groups were compared [19,23]. Only one study compared physical health state measures depending on the location of the non-union [21]. Similarly, only one study compared outcomes between infected and aseptic non-unions but there was no significant difference between the two groups. However, this could not be deemed a safe conclusion due to the small sample (*n* = 5) of infected cases [16].

### 3.6. Quantitative Analysis

Seven studies providing data that could be synthesized quantitatively were used in the pooled analysis [8,9,17,19,20,22,23]. One study [9] reported separately on tibial (TNU) and femoral non-unions (FNU) and these treatment arms were used individually in the pooling process. The rest of the studies were excluded from quantitative analysis due to the following reasons: (a) results were reported as medians and interquartile range and thus they were not suitable for quantitative synthesis [7], (b) raw data were not included [15], (c) the size of the study population was very small (six available patients to follow-up) [18], d) only mean utility scores reported which could only be used in a narrative analysis [21] and e) data were not comparable [10,16]. 

**Figure 3:**
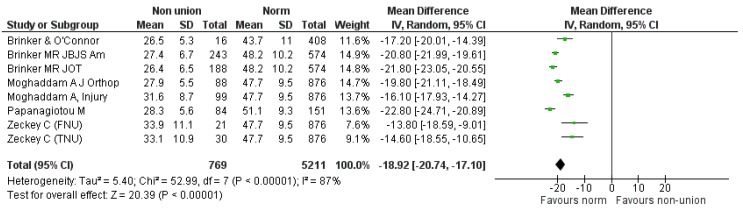
SF-12 PCS pooled analysis of non-union versus normal population.

Firstly, we compared the components of SF-12 (PCS and MCS) of the “non-union situation” against relevant “normative values” derived from age-matched populations of the same origin to the populations included in each component study [24,25]. Relevant data was derived from seven studies (eight comparisons), reporting on 769 participants [8,9,17,19,20,22,23] (Figure 3). The pooled estimate of effect size for the mean difference of PCS component between “non-union situation” and respective norms was −18.92 (95% CI: −20.74–17.1), *p* < 0.0001 (heterogeneity: I^2^ = 87%). 

**Figure 4:**
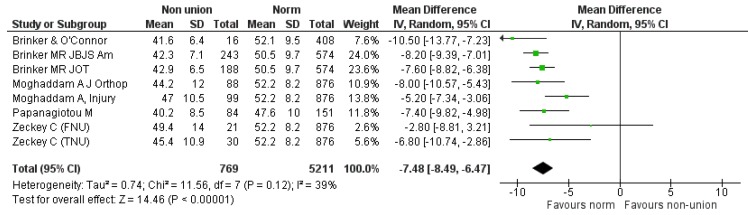
SF-12 MCS pooled analysis between non-union and respective normal population.

For the MCS component, seven studies provided relevant data [8,9,17,19,20,22,23]. Comparison was again made between 769 participants suffering from non-union of various long bones and normative values of 5211 age-matched individuals of the same origin. The summarized estimate of effect size for the mean difference of MCS component between the “non-union situation” and respective norms was −7.48 (95% CI: −8.49, −6.47), *p* < 0.0001. (Heterogeneity: I^2^ = 39%) (Figure 4). It was evident that both PCS and MCS components were significantly affected in the non-union group compared to normal population. 

**Figure 5:**
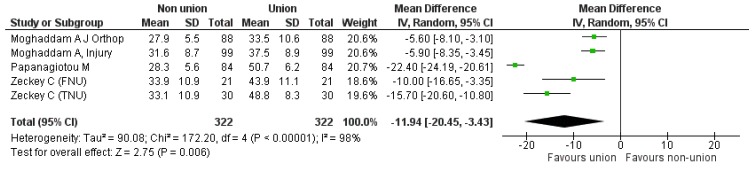
SF-12 PCS pooled analysis between nonunion and union.

**Figure 6:**
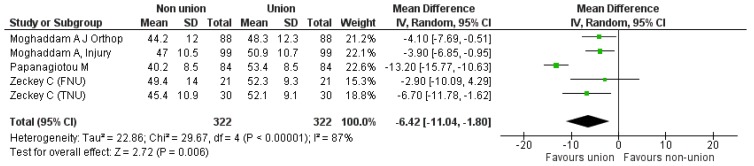
SF-12 MCS pooled analysis between nonunion and union.

Secondly, we proceeded to direct comparison of the initial “non-union” situation with the “union status” achieved at the end of a successful treatment, based on the components of the SF 12. Four studies [9,19,20,23] could be used comparing 322 cases pre- and post-intervention. Regarding the PCS component, the pooled estimate of the mean difference between non-union and union was −11.94 (95% CI: −20.45, −3.43), *p* = 0.006 (Heterogeneity: I^2^ = 98%) (Figure 5). As for the MCS component, the calculated pooled estimate of effect size favored the “bone union” situation (pooled mean difference −6.42 (95% CI: −11.04, −1.80), *p* = 0.006), (Heterogeneity: I^2^ = 87%), (Figure 6). 

### 3.7. Subgroup Analysis

**Figure 7:**
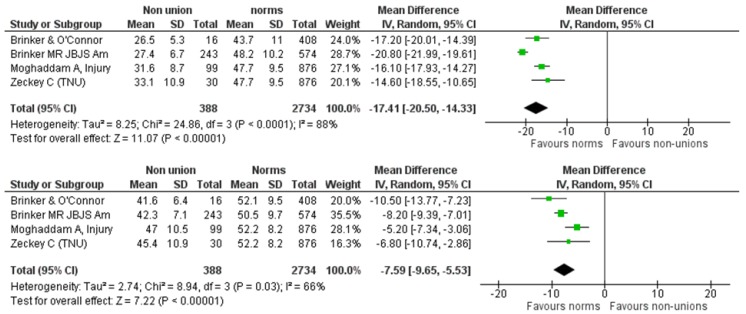
SF-12 PCS and MCS pooled analysis between tibial non-unions and norm.

**Figure 8:**
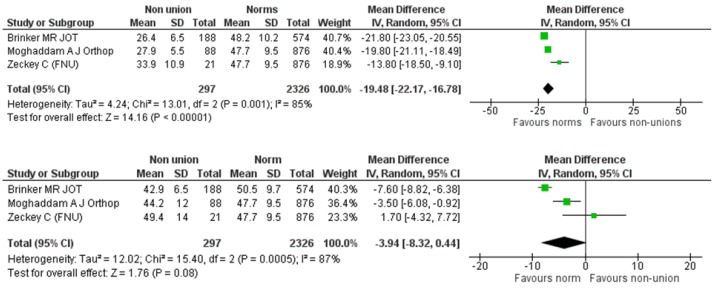
SF-12 PCS and MCS pooled analysis between femoral non-unions and norm.

We further explored the effect of the anatomical site of the non-union (femur/tibia) on the SF-12 components. We identified four eligible studies reporting on 388 participants suffering from tibial non-union [8,9,17,19]. This cohort was compared against a normative, age-matched population of 2734 individuals of the same origin with the cohorts of primary studies. The pooled estimate of the mean difference between tibial non-union and normal population was −17.41 (95% CI: −20.50, −14.33), *p* < 0.00001 (Heterogeneity: I^2^ = 88%) and −7.59 (95% CI: −9.65, −5.53), *p* < 0.00001 (Heterogeneity: I^2^ = 66%) for the PCS and MCS components, respectively (Figure 7). As for the femoral non-unions, we identified three eligible studies [9,22,23] reporting on 297 femoral non-unions. This cohort was compared against a normative, age-matched population of 2326 individuals of the same origin with the cohorts of primary studies. The summarized estimate of effect size for the mean difference of the PCS component was in favor of the normative population in a statistically significant degree (mean difference: −19.48, 95% CI: −22.17, −16.78, *p* < 0.0001, Heterogeneity: I^2^ = 85%) (Figure 8). However, the pooled estimate of effect size for the mean difference of MCS component, although favoring the normative population, did not reach levels of statistical significance: mean difference: −3.94 (95% CI: −8.32, 0.44), *p* = 0.08, I^2^ = 87% (Figure 8). 

### 3.8. Sensitivity Analysis

We repeated the pooled analysis after excluding studies with poor methodological quality. As such, there were considered studies with MINORS score of less than 12. This procedure did not yield substantially different results compared with the original ones. The results of the sensitivity analysis are depicted in Table 3. 

### 3.9. Non-Quantitative Analysis

Outcomes of interest not amenable to pooled analysis are depicted in Table 4 and Table 5. Tay et al. [7] conducted a retrospective analysis of prospective registry data over a two-year period concerning femoral and tibial shaft fractures. They used a linear regression model to compare SF-12 PCS and MCS median scores at six- and twelve-months post-injury between a group 285 united fractures and a group of 138 fractures that went on to delayed union and non-union. Patients in the union group scored higher in all categories compared to those with delayed union or non-union and these differences were statistically significant both unadjusted and adjusted for age, gender and multiple injuries. The authors also used a logistic regression model to evaluate the effect of delayed union/non-union on return to work and pain. While 72% of patients in the union group had returned to work at twelve months, only 59% of subjects with delayed union or non-union had resumed employment at the same time. This difference was statistically significant with a risk ratio of 0.82 and 0.76 for patients in the delayed union/non-union group to return to work, unadjusted and adjusted for age, gender and multiple injuries, respectively. As for pain, a risk ratio of 1.33 and 1.37 was documented for patients in the delayed union/non-union group to complain of pain at 12 months post-injury, unadjusted and adjusted for age, gender and multiple injuries, respectively. 

Wichlas et al. [10] assessed the long-term quality of life after successful surgical treatment of tibial non-unions, using SF-36 physical health status questionnaire. QoL was significantly reduced compared with normal general population in all components of the SF-36 instrument except from pain. Moreover, pain intensity and limited ankle dorsiflexion were significantly correlated with inferior QoL.

Bowen et al. [15] assessed the quality of life in a cohort of 15 patients suffering from tibial septic nonunions at a mean of 3 years following successful surgical treatment (bony excision, microsurgical soft tissue coverage and bone grafting). Nine out of the initially recruited 15 patients completed the SF-36 questionnaire and this group was compared against the age-matched United States national norms. The following subscales of the SF-36 in the treatment population were found to be significantly reduced as compared to the normative values: bodily pain, mental health, general health (at *p* < 0.001) and physical functioning (at *p* < 0.01). 

Zlowodzki et al. [16] compared the physical health state (based on SF-36 of a cohort) of 21 patients suffering from femoral or tibial non-union with that at one year post-operatively, following successful operative treatment, and also with US population normative values. The physical functioning, physical role and social functioning subscores comparing pretreatment and posttreatment values were statistically significant (*p* < 0.05). However, the posttreatment values for all subscales of the SF-36 were significantly lower than those of the normal US population (*p* < 0.01).

Braly et al. [18] assessed the physical health state in a group of six patients at an average of 4.4 years following successful treatment with percutaneous autologous bone marrow injection. Both the AAOS Lower Limb Core and SF-12 PCS scores were found to be significantly reduced at the time of the non-union situation compared with the final follow-up after successful treatment. 

Schottel et al. [21] utilized the Time Trade-Off direct measure to compute utility scores (ranging from 0.0 to 1.0, value 1.0 implying perfect health) in order to evaluate the physical health state (HRQoL) in a cohort of 832 long bone non-unions. The computed utility score for the entire non-union cohort (0.68) was even lower than that of illnesses such as type-I diabetes mellitus, stroke and acquired immunodeficiency syndrome.

Lastly, Zeckey et al. [9] investigated the impact of tibial or femoral non-unions on the potential development of anxiety or depression, using the Hospital Anxiety and Depression Scale (HADS), and Posttraumatic Stress Disorder Syndrome (PTSD), using the Impact of Event Scale (IES). They found that while femoral non-unions caused no significant psychological impairment in terms of PTSD, anxiety or depression, tibial non-unions were responsible for the development of significant symptoms of PTSD (measured by the IES) compared with matched cases of uneventful healing. However, no differences were found for anxiety or depression syndromes (Table 6). 

## 4. Discussion

Our pooled analysis showed that the physical health state of non-unions, as measured by SF-12, is significantly worse compared to normal population both in its physical and mental components. Similar results were obtained from the subgroup analysis that investigated separately the impact of tibial and femoral non unions on the components of SF-12. However, the mean difference of femoral non-unions from the appropriate norms in terms of the mental component of the SF-12, although favoring the normal population, did not reach statistically significant levels. This fact may be due to the limited number of relevant studies for pooled analysis (three studies) with significant heterogeneity across their reported results. In addition, the reported outcomes of the primary studies not amenable to pooled analysis were in line with the results of the pooled analysis further highlighting the devastating effects of long-bone non-unions on the patients’ physical health state and their psychological wellbeing.

We could identify only one study [9] that explicitly investigated the psychological impact of a non-union. Authors used both the Hospital Anxiety and Depression Score (HADS) and Impact of Event Scale (IES). HADS is a self-assessment scale developed in 1983 and validated to investigate anxiety and depression in somatically ill patients [26,27] and IES measures the impact of traumatic life events revealing the incidence of Posttraumatic Stress Syndrome (PTSD) [28]. Zeckey et al. [9] compared aseptic femoral and tibial non-unions to matched uneventful healing and found that NU groups had higher HADS scores (depression and anxiety) but this wasn’t statistically significant. 

Tibial NU had significantly higher IES score than uneventfully tibial fractures (PTSD in tibial pseudarthroses). This finding is thought to be due to the fact that treatment of resistant tibial non-unions require repeated in-hospital stays and consecutive surgical procedures that predispose to the development of posttraumatic stress disorder syndrome (PTSD) [29,30]. There was no difference in the IES score between femoral NU and uneventfully healed fractures. Although femoral shaft fractures and subsequent non-unions indicate a significant burden of injury, nevertheless the injury severity level does not seem to exert any profound effect on the symptoms of PTSD [31].

Limitations of our study include the lack of RCTs and moderate to low methodological quality of the included studies. The most important reasons for the low level of the methodological quality of the primary studies were lack of power analysis, non-blinded assessment of the outcomes and lack of contemporary comparison groups. Furthermore, there was a lack of consistency between studies in terms of outcome measures and, thus, we had to conduct a pooled analysis using seven out of 13 primary studies. More than half of our included studies (69%) [8,9,17,19,20,22,23] utilized the same (SF-12 qol outcome measure); therefore, we used its data on our statistical analysis. The SF-12 is most widely used to assess self-reported physical health state [32]. Developed originally from the Medical Outcomes Study (MOS) 36-item Short-Form Health Survey (SF-36), it includes the same eight health domains as the SF-36 with substantially fewer questions, making it a more practical research tool. Despite having been validated for use as a population health measure [33], the combination with additional HRqol measures provides more reliable findings [34]. This indicates another limitation of our study as we used only one outcome measure in our pooled analysis. Four of the studies that took QOL measures after treatment [8,19,22,23], included the non-unions that did not unite combined with the non-unions that did unite. Therefore, in analysing the physical health state results, it may have been skewed as they did not exclude the persistent non-unions or put them in a separate group. Confounding factors like duration of treatment or mechanism of injury have not been taken into account in any of the included studies (however, mechanism is mentioned in four). 

As strengths of our study, we should acknowledge that it is a comprehensive review of the literature incorporating all existing eligible studies compatible with our inclusion criteria. The presence of publication bias, as investigated by appropriate funnel plots, is unlikely. Moreover, our results are characterized by statistically significant levels and very narrow 95% CIs. Consequently, we are confident that we have not missed reports that would have substantially altered our findings. Moreover, we generated a strict study protocol with a priori definition of the criteria for both subgroup and sensitivity analysis. The results of the latter enhance our confidence on the robustness of our study findings. We also individually analysed the main findings of those studies that were not amenable to pooled synthesis. 

Based on our study findings, we conclude that psychological support and counseling should be implemented as part of the standard care to a patient suffering from such a long-term complication like a non-union. Perhaps taking into account the limitations in available resources and cost this could be implemented in two arms as initial screening and final assessment at the end of the follow-up period. However, it is more than evident that more research is needed, especially for studies measuring psychological parameters (anxiety and depression) in this group of patients (only one study available in the literature). High-quality evidence, such as RCT using non-unions of upper and lower limbs, septic and non-infected, as well as investigating any impact of the biologic type of pseudarthrosis, can be deemed as future directions for research. Our systematic review shows the significant impact that non-unions have on physical health state and psychological wellbeing, highlights areas of potential future research and foremost invites more studies to shed light in the psychological dimension of this clinical entity.

## Figures and Tables

**Table 1:** Demographic data and baseline characteristics of the primary studies.

Study No.	Name (year) [Country]	Reference	Sample	Sex distribution	Age Range (Median)	Anatomic Location	Mechanism of Injury (Open/Closed Fracture) (Type NU)
Study 1	Bowen et al. (1996) (Canada)	[15]	9 patients Septic only	Males: 8 Females: 1	17–69 (32)	Tibial	Yes
Study 2	Zlowodzki et al. (2005) (USA)	[16]	23 patients 5 septic (not defined femur/tib)	Unknown		7 Tibial 16 femoral	No
Study 3	Brinker (2007) (USA)	[17]	23 patients 8 septic	Males: 8 Females: 15	61–92 (72)	Tibial	No (open/closed)
Study 4	Zeckey et al. (2011) (Germany)	[9]	51 patients Aseptic only	M: (TNU 21/FNU 14) F: (TNU 9/FNU 7)	Femur: (41.5) Tibia: (37.6)	21 Femoral 30 Tibial	No
Study 5	Braly et al. (2013) (USA)	[18]	11 patients Aseptic only	Males: 4 Females: 7	24–51 (40.1)	Tibial	No
Study 6	Brinker et al. (2013) (USA)	[8]	237 patients 44 septic	Males: 158 Females: 79	Males: (46.3) Females: (49.4)	243 Tibial	No (open/closed)
Study 7	Tay et al. (2014) (Australia)	[7]	138 patients delayed/nonunion	Males: 107 Females: 31	127 younger than 65 years	Femoral and Tibial	Yes (open/closed)
Study 8	Moghaddam et al. (2015) (Germany)	[19]	99 patients 35 septic	Males: 74 Females: 25	(47.4)	Tibial NU 49 (1 step) 50 (2 steps)	No (open/closed)
Study 9	Papanagiotou et al. (2015) (Greece)	[20]	84 patients 30 septic	Males: 60 Females: 24	18–81 (46)	41 Tibial 30 Femoral 10 Humeral 3 Forearm	No (Hypertrophic/atrophic)
Study 10	Schottel et al. (2015) (USA)	[21]	832 patients 106 septic	Males: 488 Females: 346	18–93 (49.6)	435 Tibias 201 Femoral 125 Humeral 33 Forearm 38 Clavicle	No
Study 11	Wichlas et al. (2015) (Germany)	[10]	64 patients 30 septic	Males: 49 Females: 15	19–78 (42.8)	Tibial	Yes (open/closed) (Hypertrophic/atrophic)
Study 12	Brinker et al. (2017) (USA)	[22]	187 patients 10 septic	Males: 102 Females: 85	Males: (42.8) Females: (55.9)	188 Femoral	No (open/closed)
Study 13	Moghaddam et al. (2017) (Germany)	[23]	88 patients 72 BMP-7 16 septic	Males: 43 Females: 45	(49.9)	Femoral One stage:41 Two stages:47	Yes (open/closed) Atrophic only

SF-12: Short Form Health Survey 12 questions; SF-36: Short Form Health Survey 36 questions; WOMAC: Western Ontario and McMaster Universities Osteoarthritis Index; QALY: Quality-adjusted life year; NU: Non-union; DU: Delayed union.

**Table 2:** Study design, outcome measures, relevant groups and quality.

Author (year)	Design	QoL	Psych	Pre-Intervention	Post-Intervention	Comparison/Groups	MINORS Score
Bowen et al. (1996)	Retrospective cohort	SF-36 WOMAC	N/A		✓ (1–6 years, median 3)	Septic NU vs. USA age-adjusted norms (35–44). Absence of control group	13
Zlowodzki et al. (2005)	Prospective cohort	SF-36	N/A	✓ (2 weeks before surgery)	✓ (236–740 days, Median: 449)	vs. normal US population. Also septic vs. aseptic NU	13
Brinker (2007)	Prospective cohort	SF-12 QALY’s	N/A	✓	✓ (18–61 months, Median 38 months)	Pre-op vs. post-op outcome scores	9
Zeckey et al. (2011)	Retrospective cohort	SF-12	HADS IES		✓	aseptic NU vs. uneventful healing	10
Braly et al. (2013)	Prospective cohort	SF-12 Time trade-off	N/A	✓	✓ (1.3–8.2 years, median 4.4 years)	Pre-op vs. post-op outcome scores	12
Brinker et al. (2013)	Prospective cohort	SF-12 Time trade-off	N/A	✓		NU vs. other orthopaedic conditions/chronic medical problems/general US population	9
Tay et al. (2014)	Retrospective cohort	SF-12	N/A		✓ (up to 1 year)	NU/DU vs. uneventful healing	14
Moghaddam et al. (2015)	Prospective cohort	SF-12	N/A	✓	✓ (up to 1 year)	1-step vs. 2-step Masquelet Each group pre-op vs. post-op	17
Papanagiotoy et al. (2015)	Prospective cohort	SF-12	N/A	✓	✓ (up to 1 year)	Pre-op vs. post-op outcome scores	10
Schottel et al. (2015)	Retrospective cohort	Time trade-off	N/A	✓		NU in different anatomical sites vs. general population/medical problems	14
Wichlas et al. (2015)	Retrospective cohort	SF-36	N/A		✓ (median 5 years)	vs. normal population	16
Brinker et al. (2017)	Retrospective cohort	SF-12 Time trade-off	N/A		✓	NU vs. other orthopaedic conditions/chronic medical problems/general US population	12
Moghaddam et al. (2017)	Prospective case series	SF-12	N/A	X	X (up to 1 year)	1-step vs. 2-step Masquelet Each group pre-op vs. post-op	20

Abbreviations: SF-12: Short Form Health Survey 12 questions; SF-36: Short Form Health Survey 36 questions; WOMAC: Western Ontario and McMaster Universities Osteoarthritis Index; QALY: Quality-adjusted life year; NU: Non-union; DU: Delayed union.

**Table 3:** Results of the sensitivity analysis. (Studies with MINORS score below 12 were excluded from the pooled analysis).

Comparison	Original Analysis	Number of Studies	Refs	Mean Difference [95% CI]	Statistical Method	Statistical Model	Hetero-Geneity
SF-12 PCS pooled analysis of non-union versus normal population.	Figure 3	3	[19,22,23]	−19.30 [−22.27, −16.33]	Inverse Variance (IV)	Random effects	I^2^ = 92%
SF-12 MCS pooled analysis between non-union and respective normal population.	Figure 4	3	[19,22,23]	−6.98 [−8.57, −5.40]	IV	Random effects	I^2^ = 52%
SF-12 PCS pooled analysis between nonunion and union.	Figure 5	2	[19,23]	−5.75 [−7.50, −4.0]	IV	Fixed effects	I^2^ = 0
SF-12 MCS pooled analysis between nonunion and union.	Figure 6	2	[19,23]	−3.98 [−6.26, −1.70]	IV	Fixed effects	I^2^ = 0

**Table 4:** Outcomes of the non-pooled analysis.

Author [ref]	Wei-Han Tay [7]		Braly HL [18]		Schottel PC [21]
Anatomical Site	Fem/Tibia				Long bones
	DU/NU	P-treat	DU/NU	P-treat	Non unions
Population Size	285	138	6	6	832
SF-12					
PCS	32 (median)	44 (median)	29.5 (mean)	46.6 (mean)	
	18 (IQR)	22 (IQR)	3.3 (sd)	4.8 (sd)	
MCS	51 (median)	56 (median)	nr	nr	
	19 (IQR)	13 (IQR)			
Return to Work	59% (62/105)	72% (145/202)	nr	nr	
Ongoing Pain	72% (76/106)	54% (114/212)	nr	nr	
AAOS Lower Limb Core Scale			55.9 (40.8–83.6)	87.7 (65.7–100)	
BPI (intensity)			2.9	2.7	
BPI (intereference)			4.6	2.3	
Time Trade-off			19%	5%	Mean utility score: 0.68
Time Trade-off (years)				5.6	

BPI: Brief Pain Inventory.

**Table 5:** Results of the SF-36 in the non-pooled analysis.

Author [ref]	Wichlas F [10]			Zlowodzki M [16]			Bowen CVA [15]	
**Anatomical Site**	Tibia			Femur:16, Tib.: 7			Septic tibial nonunions (*n* = 8)	
**Population Size**	64			23			8	
SF-36	Study population (*n* = 64)	Normal population (45–65 years)	*p*-value	Study Population	After Tx	*p*-value	Study population (*n* = 8)	Normal population (35–44 years). (Only *p*-values are reported)
General Health	61 ± 25	62.7 ± 18.8	0.59	56 ± 26	57 ± 24	0.751	*p* < 0.001	
Physical Functioning	64 ± 31	79.1 ± 22.4	<0.001	232 ± 6	43 ± 29	0.002	*p* < 0.01	
Role Physical	64 ± 45	74 ± 37.6	0.08	3 ± 8	36 ± 37	0.001	ns	
Role Emotional	74 ± 43	84.8 ± 28.5	0.049	28 ± 44	49 ± 45	0.072	ns	
Social Functioning	76 ± 27	83 ± 22	0.04	27 ± 27	46 ± 32	0.042	ns	
Bodily Pain	65 ± 30	58.5 ± 25.8	<0.001	23 ± 19	28 ± 17	0.308	*p* < 0.001	
Vitality	51 ± 22	57.7 ± 18.7	0.018	42 ± 23	45 ± 22	0.681	ns	
Mental Health	64 ± 25	68.8 ± 18.5	0.13	57 ± 23	63 ± 22	0.302	*p* < 0.001	

**Table 6:** The impact of tibial and femoral non unions on the development on anxiety and depression.

Author [ref]	Zeckey C et al. [9]		
Parameter	TNU (*n* = 30)	TH (*n* = 30)	*p*-Value
IES	19.1 ± 2.5	12.7 ± 2.9	0.01
HADS-D	4.1 ± 4.1	2.8 ± 4.1	0.3
HADS-A	6.2 ± 3.7	4.8 ± 4.4	0.8
	FNU (*n* = 21)	FH (*n* = 21)	
IES	16.5 ± 11.1	18.3 ± 8.4	0.7
HADS-D	5.9 ± 4.7	3.6 ± 3.9	0.4
HADS-A	5.7 ± 4.8	3.9 ± 2.2	0.5

TNU: tibial non-unions, TH: tibiae healed, FNU: femoral non unions, FH: femora healed, IES: Impact of Event Scale, HADS: Hospital Anxiety and Depression Scale (D: Depression, A: Anxiety).
